# Concurrent peritonsillar abscess and poststreptococcal reactive arthritis complicating acute streptococcal tonsillitis in a young healthy adult: a case report

**DOI:** 10.1186/s12879-015-0780-8

**Published:** 2015-02-07

**Authors:** Elżbieta Mazur, Ewa Czerwińska, Aneta Grochowalska, Maria Kozioł-Montewka

**Affiliations:** Medical Microbiology Department, Medical University of Lublin, ul. Chodźki 1, 20-093 Lublin, Poland; Department of Otolaryngology, Regional Specialist Hospital in Radom, ul. Aleksandrowicza 5, 26-617 Radom, Poland; Microbiological Laboratory at the Regional Specialist Hospital in Radom, ul. Aleksandrowicza 5, 26-617 Radom, Poland

**Keywords:** *Streptococcus pyogenes*, Tonsillitis, Peritonsillar abscess, Poststreptococcal reactive arthritis, Antibiotic therapy

## Abstract

**Background:**

*Streptococcus pyogenes* is responsible for 5-15% and 20-30% of acute pharyngitis/tonsillitis in adults and children, respectively. It not only causes acute illness but also can give rise to local suppurative complications such as peritonsillar abscess as well as trigger the postinfectious syndromes of glomerulonephritis, acute rheumatic fever and poststreptococcal reactive arthritis. Here, we report a case of a young healthy adult in whom both peritonsillar abscess and poststreptococcal reactive arthritis developed as a complication of acute streptococcal tonsillitis. To the best of our knowledge, such a coincidence of poststreptococcal sequelae has not been reported previously.

**Case presentation:**

A 32-year-old previously healthy woman was diagnosed with acute tonsillitis by her family doctor and treated empirically with amoxicillin/clavulanic acid (875/125 mg) twice daily for 5 days. Four days after completing antibiotic therapy, peritonsillar abscess of left tonsil developed. Needle aspiration followed by incision and drainage were performed by otolaryngologist at the Emergency Department. Next, the patient was discharged home on a 10-day course of cefuroxime and metronidazole. The symptoms of peritonsillar abscess were subsiding during treatment, however on the last day of antibiotic therapy, swelling and pain of the left ankle appeared. Five days later the patient was consulted by rheumatologist. Cultures of throat swabs and abscess aspirate collected 2 weeks before revealed the presence of *Streptococcus pyogenes*. Antistreptolysin O (ASO) titer was evaluated and proved to be 412 IU/ml (normal 0–200 IU/ml). The level of C-reactive protein was 13,0 mg/L (normal <5,0 mg/L). There was no known cardiac involvement. Poststreptococcal reactive arthritis was diagnosed. Left ankle arthralgia persisted for about 5–6 weeks. Six months after the presentation at the Emergency Department, the patient was well, with ASO titer reaching 262 IU/ml.

**Conclusions:**

Clinicians should be aware that appropriate choice of antibiotic, proper dose as well as duration of therapy of acute GAS pharyngitis/tonsillitis are crucial to prevent poststreptococcal sequelae.

## Background

*Streptococcus pyogenes* (group A streptococcus, GAS) is responsible for 5 to 15% and 20 to 30% of acute pharyngitis/tonsillitis in adults and children, respectively. It not only causes acute illness but also can give rise to local suppurative complications such as peritonsillar abscess (PTA) as well as trigger the postinfectious syndromes of glomerulonephritis, acute rheumatic fever (ARF) and poststreptococcal reactive arthritis (PSRA) [1-3]. PTA, defined as a collection of pus located between the tonsillar capsule and the pharyngeal constrictor muscle, occurs mainly in young adults [4,5]. PSRA is defined as arthritis associated with proven streptococcal infection but not fulfilling the modified Jones criteria for the diagnosis of acute rheumatic fever [2,3]. Here, we report a case of a young healthy adult in whom both, PTA and PSRA, developed as a complication of acute streptococcal tonsillitis. To the best of our knowledge, such a coincidence of poststreptococcal sequelae has not been reported previously.

## Case presentation

A 32-year-old woman was diagnosed with acute tonsillitis by her family doctor. Microbiological examination was not performed at that time. Amoxicillin/clavulanic acid 875/125 mg twice daily for 5 days was prescribed empirically. The symptoms of tonsillitis resolved within five-day treatment, however, four days after completing the course of antibiotic therapy, sore throat, more prominent on the left side, reappeared. Two days later the patient presented to the Emergency Department with a two-day history of worsening sore throat, painful swallowing and fever. The patient was previously well, with no history of chronic diseases, recurrent tonsillitis or previous peritonsillar abscess. Seven months previously she gave birth to her second child and was still breastfeeding the baby. She denied smoking. On physical examination her temperature was 38°C, pulse rate: 80 beats/min, respiratory rate: 22 breaths/min and blood pressure: 120/80 mm Hg. Examination of the oral cavity and oropharynx showed enlarged and inflamed left tonsil as well as congested and bulging soft palate on the left side with contralateral displacement of the uvula. Both tonsils were covered with whitish exudate. No dental caries was noted. There was also bilateral, moderately tender submandibular lymphadenopathy. The remainder of physical examination was unremarkable. Blood tests results are shown in Table 1. Separate swabs were obtained from the surfaces of both tonsils. Next, under local anesthesia, diagnostic needle aspiration of left tonsil was performed by otolaryngologist, during which scanty amount of pus was obtained. This initial procedure was followed by incision and drainage. Tonsil swabs and abscess aspirate were sent to laboratory for microbiological examination. The patient refused hospitalization at the Otolaryngology Department, thus was discharged home on a 10-day course of cefuroxime (500 mg twice daily) and metronidazole (500 mg 3 times daily) with recommendation to discontinue breastfeeding for the duration of the antibiotic therapy and to present at follow-up visit to Otolaryngology Clinic after completing antibiotic therapy. The symptoms of peritonsillar abscess as well as fever were subsiding steadily during treatment, however on the last day of antibiotic therapy, swelling and pain of the left ankle appeared, thus the patient presented to her family doctor. Upon presentation she was afebrile and had marked oedema and pain of her left ankle. Her heart rate was 72 beats/min, she did not have an appreciable cardiac murmur. Her chest was clear to auscultation. Antibiotic therapy with cefuroxime (500 mg once daily) for next 5 days was prescribed, as well as pain relief medication with paracetamol. After completing antibiotic therapy the patient presented at follow-up visit to Otolaryngology Clinic. She was afebrile, with normal vital signs. Examination of oropharynx showed resolution of both, congestion and oedema of left tonsil and soft palate. There was no exudate on the tonsils. Cultures of throat swabs and abscess aspirate collected 2 weeks before revealed the presence of *Streptococcus pyogenes* in all three materials. According to the patient report, left ankle swelling with which she presented to her family doctor, resolved within 4 days. Upon presentation the patient only had moderate pain in the joint. She was consulted by rheumatologist. Her heart rate was 70 beats/min. She did not have a regurgitant murmur. Her chest was clear to auscultation. Antistreptolysin O (ASO) titer was evaluated and proved to be 412 IU/ml (normal 0–200 IU/ml). The level of C-reactive protein was 13,0 mg/L. Control throat swabs were collected for culture, which revealed normal oropharynx flora. The patient was recommended to continue pain relief medication with paracetamol and present at follow-up visit to Rheumatology Clinic after 2 weeks. Her only complaint was persisting slight pain in left ankle joint. The results of physical examination were analogous to those observed at previous follow-up. ASO titer was 503 IU/ml. Control throat swabs were collected and culture yielded normal flora. The patient was recommended to present at follow-up visit at Rheumatology Clinic after 2 months. At that time, she was well. Ankle pain, according to the patient report, disappeared shortly after last visit. The results of physical examination were analogous to those observed previously. ASO titer was 396 IU/ml. Six months after the presentation at Emergency Department, the patient was well, with ASO titer reaching 262 IU/ml. Table 1 summarizes the chronology of clinical findings and blood tests results.

**Table 1:** **Summary of clinical findings and blood tests over time**

**Timeline**	**Clinical findings**	**Blood tests results**
Day 1: presentation to the family doctor	Acute tonsillitis	Not performed
Day 11: presentation to the Emergency Department	Left side peritonsillar abscess	Leukocytosis: 15 270/mm^3^ Granulocytes: 79.7%
		Lymphocytes: 15.9%
		Monocytes: 2.8%
		Hemoglobin: 13.0 g/dl Hematocrit: 38.9%
		Platelet count: 329 000/mm^3^
		CRP: 131.0 mg/L (normal <5.0 mg/L)
Day 20: 2nd presentation to the family doctor	Left ankle arthritis	Not performed
Day 25: follow-up visit to the Otolaryngology Department and the rheumatologist consultation	Left ankle arthralgia	ASO: 412 IU/ml (normal 0–200 IU/ml)
		CRP: 13.0 mg/L
Day 40: 1st follow up to the rheumatologist	Left ankle arthralgia	ASO: 503 IU/ml
Day 100: 2nd follow up to the rheumatologist	Healthy	ASO: 396 IU/ml
Day 180: 3rd follow up to the rheumatologist	Healthy	ASO: 262 IU/ml

CRP: C-reactive protein; ASO: antistreptolysin titer.

### Bacteriology findings

Cultures of PTA aspirate revealed *Streptococcus pyogenes* as a predominant species as well as *Prevotella oralis* and *Haemophilus parainfluenzae*. Tonsil swabs, collected at the time the patient presented with PTA, yielded copious growth of *Streptococcus pyogenes* as well as normal throat flora, namely *Streptococcus viridians, Neisseria spp*, and *Haemophilus parainfluenzae*. Two control throat swabs revealed only normal oropharynx flora. Bacterial species were identified with the use of routine microbiological methods, drug susceptibility of *S. pyogenes* was assessed using Vitek 2 Compact (bioMérieux). Antimicrobial susceptibility results were interpreted according to the European Committee on Antimicrobial Susceptibility Testing recommendations (EUCAST 2013, version 3.1) [6]. *Streptococcus pyogenes* strains isolated from tonsil swabs and abscess aspirate demonstrated identical susceptibility patterns. They were resistant to erythromycin, clindamycin, tetracycline and fully susceptible to all other antibiotics tested. MLS_i_ phenotype (inducible coresistance to macrolide, lincosamide and streptogramine) was detected with the use of double-disc diffusion testing [7].

## Discussion

GAS pharyngitis is a self-limiting disease in most cases, however, it can cause suppurative and nonsuppurative complications [1,2]. In our patient both, peritonsillar abscess and poststreptococcal reactive arthritis occurred as a complication of acute GAS tonsillitis. Although microbiological examination was not performed at the time the patient presented with tonsillitis, the reappearance of sore throat within 4 days after completing a 5-day antibiotic therapy with amoxicillin/clavulanic acid and the presence of GAS in throat swab cultures at the time she presented with PTA, strongly suggest GAS aetiology of antecedent tonsillitis. Moreover, the isolation of GAS strains with identical susceptibility patterns from both, throat swabs and abscess aspirate, leaves no doubt that GAS strain that caused tonsillitis participated in the development of PTA. From the majority of PTA aspirates polymicrobial mixture of aerobic and anaerobic bacteria is recovered, however, GAS along with *Fusobacterium necrophorum* are commonly regarded to be the primary pathogens [4,5]. In our patient three bacterial species were detected in abscess aspirate: GAS, *Prevotella oralis*, and *H. parainfluenzae*. There are no uniform recommendations regarding PTA antibiotic therapy, thus treatment options vary greatly between clinicians and are based mainly on their preferences [4,8,9]. In most cases antibiotics of choice include penicillin combined with metronidazole, amoxicillin with clavulanic acid, clindamycin, cefuroxime, or metronidazole [8-10]. In our department cefuroxime combined with metronidazole is administered as empiric antimicrobial therapy in most cases, our patient was treated with these drugs as well.

PSRA is defined as arthritis associated with proven streptococcal infection but not fulfilling the modified Jones criteria for the diagnosis of acute rheumatic fever (ARF). It is still not clear whether this entity represents a distinct syndrome or is a manifestation of ARF [2,3]. ARF has now become rare in developed countries. Its incidence in Western Europe is currently less than 1 case per 100 000 population, whereas PSRA is relatively more frequent with the annual incidence of approximately 2 cases per 100 000 people [11]. There is a mean interval of 14 days between the onset of GAS pharyngitis symptoms and the occurrence of PSRA [3]. Age distribution appears to be bimodal, with two incidence peaks, at ages 8–14 and 21–37 years, respectively [3]. Joint involvement is typically non-migratory and affects large joints, particularly those of lower limbs. Knee and ankle joints are regularly involved, although small joints and axial involvement occurs as well. Mono-, oligo- and polyarthritis are equally represented. Unlike the self-limiting and exquisitely responsive to salicylates arthritis of ARF, PSRA responds relatively poorly to salicylates and nonsteroid anti-inflammatory drugs. Carditis is a rare event. The disease resolves within weeks [3,11,12]. Discrimination between ARF and PSRA is ambiguous due to the lack of generally accepted set of criteria for the diagnosis of PSRA [11]. In our patient non-migratory monoarthritis, localized in the left ankle, without fever or known cardiac involvement occurred approximately 20 days after the onset of tonsillitis while the patient was still on antibiotic therapy due to PTA. Although joint oedema resolved within 4 days, arthralgia, moderately responsive to nonsteroid anti-inflammatory drugs, persisted for about 5–6 weeks. Antecedent GAS throat infection was confirmed by cultures as well as serologically. However, the modified Jones criteria were not fulfilled, thus PSRA was diagnosed. Similarly to what was noted by Jansen et al. [13], clinical findings of PSRA in our patient had subsided before ASO titre reached its maximum value. At present, there are no evidence-based guidelines whether patients with PSRA, similarly to those with ARF, should receive long-term antibiotic prophylaxis [11]. Recent data indicate no increased risk of valvular heart disease in adults with PSRA [14]. Accordingly, our patient did not receive secondary antibiotic prophylaxis.

Some European guidelines, among them British, Scottish, Dutch and Belgian, consider GAS pharyngitis to be a mild, self-limiting disease that does not require a specific diagnosis or antimicrobial treatment except in high-risk patients, such as those with a history of valvular heart disease or acute rheumatic fever, immunosuppressed or severely ill [15-18]. Recently issued recommendations of European Society of Clinical Microbiology and Infectious Diseases represent similar approach [19]. However, the remaining European and North American guidelines recommend that all cases of acute streptococcal pharyngitis/tonsillitis should be appropriately treated to prevent both, suppurative and nonsuppurative poststreptococcal complications [20]. According to Polish recommendations, phenoxymethyl penicillin, 2–3 million units twice daily for 10 days, is currently antibiotic therapy of choice for GAS pharyngitis. Second and third-line drugs include: first generation cephalosporin in patients with penicillin allergy who do not have immediate hypersensitivity to beta-lactam antibiotics or macrolides (erythromycin, azithromycin, clarithromycin) in those with hypersensitivity to beta-lactam antibiotics. Azithromycin is the only drug that is given in a 5-day course as opposed to a 10-day course for all the other antibiotics [21]. Currently, American Heart Association/American Academy of Pediatrics and Infectious Diseases Society of America recommend amoxicillin once or twice daily for 10 days as alternative first-line therapy, since in comparative clinical trials once-daily amoxicillin (50 mg/kg, to a maximum of 1000 mg) for 10 days has been shown to be effective for GAS pharyngitis [2,22]. However, the treatment of tonsillitis in our patient did not comply the recommendations, particularly with respect to the duration of therapy. Five-day treatment with amoxicillin/clavulanic acid resulted in the lack of GAS eradication, which in turn caused both PTA and PSRA.

## Conclusion

In summary, to the best of the authors’ knowledge, this is the first published account of the coincidence of both PTA and PSRA, complicating acute GAS tonsillitis in a young healthy adult. Moreover, the occurrence of both complications might have an association with improper management of acute streptococcal tonsillitis. Clinicians should be aware that appropriate choice of antibiotic, proper dose as well as duration of therapy of acute GAS pharyngitis/tonsillitis are crucial to prevent poststreptococcal sequelae.

## Consent

Written informed consent was obtained from the patient for publication of this case report. A copy of the written consent is available for review by the Editor of this journal.
